# Spatially explicit ecological modeling improves empirical characterization of plant pathogen dispersal

**DOI:** 10.1002/pei3.10104

**Published:** 2023-04-09

**Authors:** Petteri Karisto, Frédéric Suffert, Alexey Mikaberidze

**Affiliations:** ^1^ Plant Pathology Group, Institute of Integrative Biology ETH Zurich Zurich Switzerland; ^2^ Plant Health Natural Resources Institute Finland Jokioinen Finland; ^3^ Université Paris‐Saclay, INRAE, AgroParisTech, UMR BIOGER 78850 Thiverval‐Grignon France; ^4^ School of Agriculture, Policy and Development University of Reading Whiteknights Reading UK

**Keywords:** dispersal ecology, dispersal gradient, dispersal kernel, dispersal theory, experimental design, mathematical modeling

## Abstract

Dispersal is a key ecological process, but it remains difficult to measure. By recording numbers of dispersed individuals at different distances from the source, one acquires a dispersal gradient. Dispersal gradients contain information on dispersal, but they are influenced by the spatial extent of the source. How can we separate the two contributions to extract knowledge about dispersal? One could use a small, point‐like source for which a dispersal gradient represents a dispersal kernel, which quantifies the probability of an individual dispersal event from a source to a destination. However, the validity of this approximation cannot be established before conducting measurements. This represents a key challenge hindering progress in characterization of dispersal. To overcome it, we formulated a theory that incorporates the spatial extent of sources to estimate dispersal kernels from dispersal gradients. Using this theory, we re‐analyzed published dispersal gradients for three major plant pathogens. We demonstrated that the three pathogens disperse over substantially shorter distances compared to conventional estimates. This method will allow the researchers to re‐analyze a vast number of existing dispersal gradients to improve our knowledge about dispersal. The improved knowledge has potential to advance our understanding of species' range expansions and shifts, and inform management of weeds and diseases in crops.

## Graphical abstract

Plants and their pathogens produce offspring that needs to move (or disperse) to a different location to be able to survive and reproduce further. It is important to know how far they can move because this would inform ways to protect endangered species and manage weeds and diseases in crops. To measure dispersal, we typically record the numbers of dispersed individuals at different distances from the source. However, since the source itself can have a substantial area, we may not know where exactly each individual originated, and this introduces a large uncertainty into measurements. We have overcome this problem by formulating a mathematical theory of dispersal that sums over all dispersal events across the entire source area and thereby makes dispersal measurements much more accurate. Using this theory, we demonstrated that three important pathogens of major crops (wheat and potato) disperse over substantially shorter distances than previously thought. This means that they will shift or expand the geographic areas where they cause problems slower than expected.

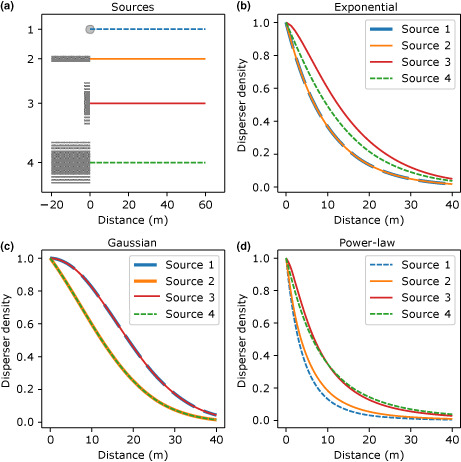

## 1 INTRODUCTION

Dispersal is an important component of many life histories. Knowledge of dispersal is crucial to our understanding of fundamental population biological processes, e.g., changes in species' geographic ranges (Bonte & Dahirel, 2017; Brooker et al., 2007; Kubisch et al., 2013) as well as size and composition of ecological communities (Leibold et al., 2004; Ron et al., 2018). This knowledge also improves our capacity to conserve endangered species (McConkey et al., 2012; Musciano et al., 2020) and protect crop plants from weeds (Petit et al., 2013) and diseases (Fabre et al., 2012; Mikaberidze et al., 2016). Consequently, empirical characterization of dispersal has been a major research theme for over a hundred years (e.g., Bullock et al., 2017; Fabre et al., 2021; Heald, 1913; Nathan et al., 2012). However, there is still far fewer datasets describing plant dispersal than plant demography because dispersal remains difficult to measure (Bullock et al., 2017). Here, we identified and resolved one of the key challenges that hinders progress in empirical characterization of dispersal: We incorporated the spatial extent of dispersal sources in the analysis of dispersal measurements.

One approach to measure dispersal is to use spatially localized sources of dispersing individuals and record dispersal gradients produced from them. These gradients do contain relevant information about dispersal, but they are also influenced by the spatial extent of the source (Cousens & Rawlinson, 2001; Ferrandino, 1996; Zadoks & Schein, 1979).

How can we evaluate this influence to extract more general knowledge about dispersal from specific dispersal gradients? This can be achieved using a mathematical description of dispersal with the help of dispersal kernels. A dispersal kernel quantifies the probability of an individual dispersal event from a source point to a destination point. Technically, a dispersal kernel is a probability density function that depends on the location of the destination point (“dispersal location kernel”, Nathan et al., 2012).

To characterize dispersal, we need to estimate dispersal kernels based on observed dispersal gradients. An observed dispersal gradient from a point source would correspond to the dispersal kernel. However, sources usually need to have a certain area to yield sufficient number of dispersing propagules to be observed. How can we achieve a sufficiently small source to be considered a point? In an influential book on plant disease epidemics, Zadoks and Schein (1979) formulated a rule of thumb stating that a point source should have “a diameter smaller than 1% of the gradient length”. However, this rule of thumb is misleading. The size of the source should be compared with the characteristic distance of dispersal rather than the gradient length. However, we do not know the characteristic dispersal distance in advance of conducting measurements. Therefore, we cannot establish sound criteria for the validity of the point source approximation in advance of conducting measurements.

Due to the lack of clear criteria, “point” sources of various sizes appear in the literature: an adult tree (lichen *Lobaria pulmonaria*, Werth et al., 2006), circles of 1.6 m diameter (thistles *Carduus nutans, Carduus acanthoides*, Skarpaas & Shea, 2007), circles of 0.5 m diameter (garlic mustard *Alliaria petiolata*, Loebach & Anderson, 2018), 4 m^2^ squares (wine raspberry *Rubus phoenicolasius*, Japanese barberry *Berberis thunbergii*, multiflora rose *Rosa multiflora*, and Japanese stiltgrass *Microstegium vimineum*, Emsweller et al., 2018), and even entire agricultural fields (oilseed rape *Brassica napus*, Devaux et al., 2007). These studies reported valuable dispersal gradients, but using these dispersal gradients as proxies for dispersal kernels may be unjustified. Spatially explicit modeling has been suggested (Greene & Calogeropoulos, 2002) to address this problem and was used in some modeling studies (Clark et al., 1999; Shaw et al., 2006), but it was not widely adopted in the literature on experimental dispersal measurements.

In this study, we devised a systematic approach to estimate dispersal kernels from dispersal gradients without using the point source approximation. For this purpose, we combined theory, analysis of empirical data and numerical simulations. We first formulated a theory that incorporates dispersal from a spatially extended source considering each point within the source area as an independent point source (the spatially explicit approach). We highlighted how mathematical properties of widely used kernel functions (exponential, Gaussian and power‐law, Nathan et al., 2012) can inform experimental design. Then, we re‐analyzed published empirical datasets on three major plant pathogens with contrasting spatial scales of dispersal and conducted comprehensive numerical simulations. In this way, we demonstrated how this approach allows the researchers to estimate dispersal kernels more accurately than using the point source approximation.

## 2 DESCRIPTION

### 2.1 Theory

The probability of dispersal from a source point $p_{s}=\left(x_{s},y_{s}\right)$ to a destination point $p_{d}=\left(x_{d},y_{d}\right)$ is given by the *dispersal location kernel* (hereafter “dispersal kernel”). It is typically a monotonically decreasing function of the distance between the points.

To estimate a dispersal kernel using a dispersal gradient produced by an area source, we consider the cumulative effect of all point‐to‐point dispersal events from the source to the destination. This is achieved by taking an integral over the individual points comprising the source to calculate their combined contribution to the dispersed population at a certain destination point (as in Shaw et al., 2006, Equation (4.6)). Similarly, the integral over all points of the destination area gives the total number of individuals that moved there from the source (as in Rimbaud et al., 2018, Equation (16)):

(1) $$N_{1}\left(S,D\right)=\iint_{D}\iint_{S}n_{0}\left(p_{s}\right)\kappa \left(p_{s},p_{d}\right)\;{\mathrm{dA}}_{S}\;{\mathrm{dA}}_{D},$$

where $S=\left\{p_{s}\right\}$ is the source area, $D=\left\{p_{d}\right\}$ is the destination area, $n_{0}\left(p_{s}\right)$ is the density of individuals within S before dispersal, and $\kappa \left(p_{s},p_{d}\right)$ is the dispersal kernel (key variables and parameters are listed in Table 1). Equation (1) provides a valid description of the dispersal process when the overall population size is sufficiently large so that stochastic fluctuations in the numbers of dispersed individuals can be neglected. When the populations before dispersal ($n_{0}\left(p_{s}\right)$) and after dispersal ($N_{1}$) are measured, the only unknown in Equation (1) remains the dispersal kernel. Equation (1) offers a way to estimate dispersal kernel parameters that takes into account the spatial extent of both the source and the destination.

**TABLE 1:** Key variables and parameters.

Symbol	Definition
κ	Dispersal kernel
$n_{0}$	Population density in the source area before dispersal [$\cdot /m^{2}$]
$N_{0}$	Population size in the source area before dispersal
$N_{1}$	Population size in the destination area after dispersal
S	Source area
D	Destination area
$p_{s}$, $p_{d}$	A source point/a destination point
$C_{k,e}$/$C_{k,g}$/$C_{k,p}$	Normalization factors of exponential/Gaussian/power‐law kernels
k=1,2	Number of dimensions
$I_{0}$	Disease intensity in the source area before dispersal [$\cdot /m^{2}$]
$I_{1}$	Disease intensity in the destination area after dispersal [$\cdot /m^{2}$]
β	Transmission parameter
α	Scale parameter of exponential or Gaussian kernel [m]
γ	Shape parameter of power‐law kernel
λ	Scale parameter of power‐law kernel [m]
r	Distance from the source [m]
$\bar{r}$	Mean dispersal distance [m]
$r_{L}$	L th percentile of the dispersal distance kernel [m]
$x_{s}$/$y_{s}$	x‐/y‐coordinate of a source point [m]
$x_{d}$/$y_{d}$	x‐/y‐coordinate of a destination point [m]
$w_{x}$/$w_{y}$	Width of the source area along the x‐axis/y‐axis [m]
$w_{d}$	Width of the destination area along the y‐axis [m]
$b_{s}$/$b_{d}$	Width of the border zone outside the source area/destination area along the y‐axis [m]

A simpler, more common but often inaccurate approach is to fit a function of one spatial coordinate x to dispersal gradient data. For example, the function

(2) $$N_{1}={\mathrm{Ce}}^{-x/\alpha }$$

can be fitted to dispersal gradient data to estimate the scale parameter α of the exponential kernel (for example, Saint‐Jean et al., 2004). This approach works for any kernel function if both the source and the destination can be considered as points.

**FIGURE 1:**
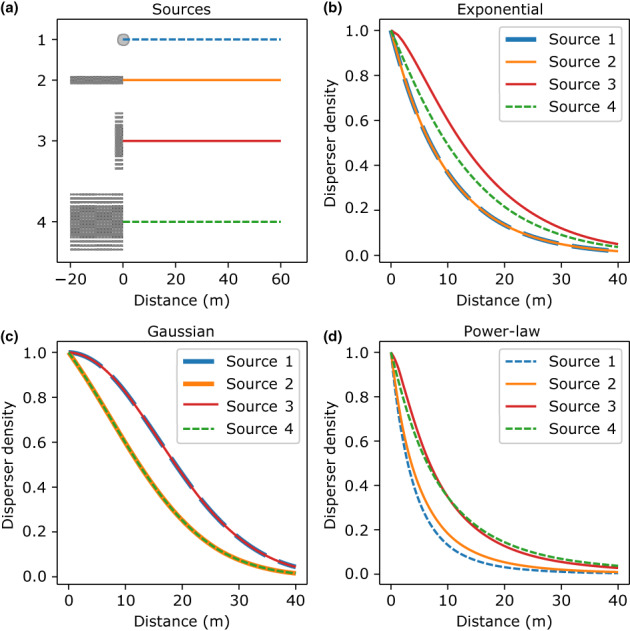
Different extensions of the source (panel a) lead to different effects on the dispersal gradients (b–d) depending on the dispersal kernel. Kernel parameters are chosen so that the mean dispersal distance $\bar{r}=20$ m in all cases. All gradients are normalized to begin at one. (a) Four different sources (gray shapes): (1) a point source; (2) a line source, parallel to the gradient (colored lines) $x_{S}\in \left[-20\;m,0\;m\right]$; (3) a line source perpendicular to the gradient, $y_{s}\in \left[-100\;m,100\;m\right]$; (4) a rectangular area source, $\left(x_{s},y_{s}\right)\in \left[-20\;m,0\;m\right]\times \left[-100\;m,100\;m\right]$. (b) With the exponential kernel (α=10 m), sources 1 and 2 result in identical gradients. (c) With the Gaussian kernel (α=16 m), the gradients are identical between sources 1 and 3 and between sources 2 and 4. (d) With the power‐law kernel (γ=5,λ=20 m) all gradients are different.

However, when the source or the destination is extended in space, the above approach may lead to inaccurate estimates of kernel parameters. In particular, extended sources modify dispersal gradients compared to point sources. Figure 1 illustrates such modifications for exponential, Gaussian and power‐law kernels (defined in Box 1). Compare, for example, the gradients produced by the point source (source 1 in Figure 1a) and the area source (source 4 in Figure 1a). Extension of the source leads to a “flattening” of the gradient for the exponential and the power‐law kernels, but for the Gaussian kernel, it leads to a “steepening” of the gradient (cf. the dashed green curve with the dashed blue curve in Figure 1b,d). Some studies postulated that gradients produced by spatially extended sources are “flatter” than gradients resulting from more localized sources (Cousens & Rawlinson, 2001; Ferrandino, 1996; Greene & Calogeropoulos, 2002; Zadoks & Schein, 1979). Here, we demonstrated that whether the extension of the source leads to a “flattening” or to a “steepening” of the gradient depends on the underlying kernel function. Thus, using a dispersal gradient from an extended source as a proxy for a dispersal kernel can lead to either an overestimation or an underestimation of the associated kernel parameters.

BOX 1 **Dispersal kernels and their special properties.**

**Exponential kernel** is defined as

(3) $$\kappa_{e}\left(r\right)=C_{k,e}e^{-r/\alpha },$$

where α is the scale parameter, $k\in \left\{1,2\right\}$ is the number of dimensions, $r=r\left(p_{s},\;p_{d}\right)$ is the Euclidean distance from the source point $p_{s}=\left(x_{s},y_{s}\right)$ to the destination point $p_{d}=\left(x_{d},y_{d}\right)$ (in one dimension $y_{s}=y_{d}=0$), and $C_{k,e}$ is the normalization factor: $C_{1,e}=1/\left(2\alpha \right)$, $C_{2,e}=1/\left(2{\pi \alpha }^{2}\right)$. The mean dispersal distance for k=2 is ${\bar{r}}_{e}=2\alpha$.

**Gaussian kernel** is defined as

(4) $$\kappa_{g}\left(r\right)=C_{k,g}e^{-r^{2}/2\alpha^{2}},$$

where α is the scale parameter, $C_{1,g}=1/\sqrt{2{\pi \alpha }^{2}}$, and $C_{2,g}=1/\left(2{\pi \alpha }^{2}\right)$. The mean dispersal distance for k=2 is ${\bar{r}}_{g}=\alpha \sqrt{\pi /2}$.

**Power‐law kernel** is defined here as

(5) $$\kappa_{p}\left(r\right)=C_{k,p}{\left(\lambda +r\right)}^{-\gamma },$$

where γ is the shape parameter, λ is the scale parameter, $C_{1,p}=\left(\gamma -1\right)\lambda^{\gamma -1}$, $C_{2,p}=\left(\gamma -2\right)\left(\gamma -1\right)\lambda^{\gamma -2}/\left(2\pi \right)$. The mean dispersal distance for k=2 is ${\bar{r}}_{p}=2\lambda /\left(\gamma -3\right)$ for γ>3 and ${\bar{r}}_{p}=\infty$ for γ≤3.

**Memorylessness.** Exponential kernels are memoryless: when we set any point in the distribution as a starting point, the tail of the distribution will have the same shape as the entire distribution, i.e., the “past” does not affect the “future” probabilities:

$$\frac{f\left(x_{1}\right)-f\left(x_{1}+y\right)}{f\left(x_{1}\right)}=\frac{f\left(x_{2}\right)-f\left(x_{2}+y\right)}{f\left(x_{2}\right)},$$

for any starting points $x_{1},x_{2}$ (see also Ahmad & Alwasel, 1999). Thanks to this property, exponential kernels are unambiguously characterized by the half‐distance $\alpha \ln\left(2\right)$. For any r‐value in Equation (3), moving $\alpha \ln\left(2\right)$ units further to $r+\alpha \ln\left(2\right)$ will decrease the density by half.

**Separability.** A function is called separable when it can be expressed as a product of other functions that depend on only one independent variable each: the variables can be separated from each other, e.g., $f\left(x,y\right)=f_{x}\left(x\right)f_{y}\left(y\right)$. Separable functions are often considered in connection with separable differential equations (Ahmad & Ambrosetti, 2015). When a dispersal kernel is separable, the shape of the kernel along the x‐axis does not depend on the y‐coordinate, i.e., dispersal probabilities in x‐ and y‐dimensions are independent random variables.

Only in special cases, does the shape of the dispersal gradient match to the shape of the dispersal kernel even when the source is extended, whereby the analysis can be simplified. (i) If the source is extended only in the direction of the measured gradient (along the x‐axis) and dispersal is governed by the exponential kernel, using Equation (2) will give a correct estimate of α, because exponential kernels are “memoryless” (Box 1). This is visible in Figure 1b where source 1 and source 2 produce the same gradients. However, this does not work for Gaussian and power‐law kernels (Figure 1c,d). (ii) If the source is extended along the y‐axis, perpendicular to the direction of measured gradient, a similar simplification is possible for the Gaussian kernel (Figure 1c, source 1 and 3). This is due to separability (Box 1) of the kernel, whereby each point source within the line source 3 in Figure 1a produces the same gradient along the x‐axis. This holds for any separable kernel, but does not hold for non‐separable kernels such as exponential or power‐law kernels (Figure 1b,d). Analogous simplifications can be made when considering spatially extended destinations.

Insights presented above inform design and analysis of dispersal experiments. Gaussian and exponential kernels have been used in a number of studies to describe dispersal across a range of taxonomic groups (Table 15.1 in Nathan et al., 2012). When dispersal is governed by a memoryless (exponential) or a separable (e.g., Gaussian) kernel, appropriate line sources should be used to boost the power of the source, while maintaining the validity of the point source approximation to simplify the analysis. However, in most cases dispersal is better described by kernels that are neither memoryless nor separable (Nathan et al., 2012), such as the power‐law kernel in Equation (5). In these cases, or when the kernel function is not known before conducting measurements, dispersal gradients should be analyzed using a spatially explicit approach based on Equation (1), as we demonstrate next.

### 2.2 Experimental design and data analysis

**FIGURE 2:**
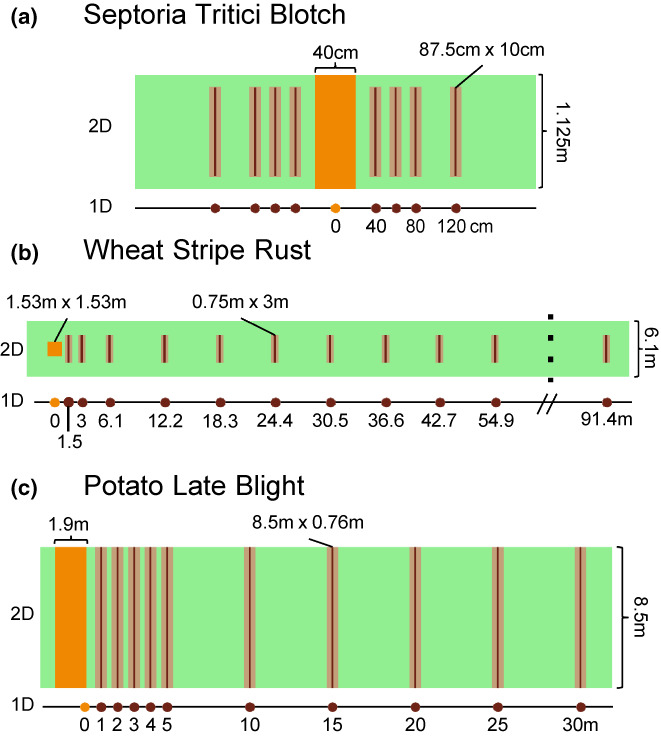
Designs of the experimental plots. (a) Septoria tritici blotch experiment (Karisto et al., 2022); (b) yellow rust experiment (Cowger et al., 2005; Sackett & Mundt, 2005); (c) potato late blight experiment (Gregory, 1968). The two‐dimensional (2D) view corresponds to the spatially explicit approach; the one‐dimensional (1D) view corresponds to the point source approximation. Inoculation areas are shown in orange, measurement lines in light brown and the approximated thin lines in dark brown.

We re‐analyzed published empirical data on dispersal gradients using the spatially explicit method that incorporates the spatial extent of the source and compared the outcomes with those based on the conventional point source approximation. We considered three datasets collected in field experiments investigating dispersal of major pathogens of crop plants: (i) the fungus *Zymoseptoria tritici* that causes septoria tritici blotch in wheat (Karisto et al., 2022); (ii) the fungus *Puccinia striiformis* that causes stripe (yellow) rust in wheat (Cowger et al., 2005; Sackett & Mundt, 2005); and (iii) the oomycete *Phytophthora infestans* that causes late blight in potatoes (Gregory, 1968). The three pathosystems represent contrasting mechanisms and spatial scales of dispersal. Asexual spores of *Z. tritici* (pycnidiospores) move pre‐dominantly via rain splash, while asexual spores of *P. striiformis* (urediniospores) and propagules of *P. infestans* (sporangia) are mainly wind‐dispersed. Spatial scales of the experiments varied from 100 cm to 100 m. Design of experimental plots and measurements is shown in Figure 2.

In each experiment, pathogen spores were inoculated across inoculation areas within experimental plots to create area sources of dispersing populations (orange areas in Figure 2). Then, disease gradients (disease intensity versus distance from the source) were recorded outside the inoculation areas across rectangular areas situated at increasing distances from the source (we call these areas “measurement lines”; light brown rectangles in Figure 2). These disease gradients are called primary gradients, because they resulted from a single cycle of pathogen reproduction (based on latent periods and timing of infections). The cycle includes both spore dispersal and infection success, hence, the measured gradients reflected effective dispersal gradients of the pathogen population (analogous to the combination of seed dispersal and establishment, Klein et al., 2013).

In the analysis, we incorporated the spatial extent of the source areas in two dimensions, but considered the measurement lines as thin lines perpendicular to the dispersal direction, since their length along the dispersal direction was short (dark brown lines in Figure 2). For each dataset, we chose an appropriate dispersal kernel function based on the original study, to allow for comparison with the results of the original analysis. Then we derived specific expressions for dispersal gradients, firstly, using the point source approximation (i.e., assuming a point‐like source and destinations in the middle of the inoculation area or measurement lines; “1D” in Figure 2) and, secondly, based on the spatially explicit Equation (1). Based on these expressions, we estimated dispersal kernel parameters.

#### 2.2.1 Septoria tritici blotch

We analyzed a subset of data collected in a larger experiment (Karisto et al., 2022) that characterized dispersal of a specific strain of *Z. tritici* (ST99CH_3D7). The plots were 1.125 m wide (y‐dimension) and 4 m long (x‐dimension). Inoculation areas were 40 cm wide in x‐dimension and spanned across entire plot in y‐dimension (Figure 2a). Disease intensity in the measurement lines was measured as the density of the fungal fruiting bodies (pycnidia) on leaves using automated digital image analysis (Karisto et al., 2018; Stewart et al., 2016).

Using the point source approximation, we computed the disease intensity after dispersal at a distance r=x from the source with exponential kernel (Equation (3), k=1) as

(6) $$I_{1}\left(x\right)=\frac{I_{0}\beta }{2\alpha }e^{-|x|/\alpha },$$

where $I_{0}$ is the disease intensity at the source before dispersal and β is the transmission parameter comprising the probability of dispersal and the infection efficiency of fungal spores.

Next, we relaxed the point source approximation and used the spatially explicit approach. We computed the expected disease intensity after dispersal at a destination point by substituting the exponential kernel with k=2 into Equation (1). We specified the integrals in Equation (1) according to the plot design across the inoculation area (source; $w_{x}=0.4$ m × $w_{y}=1.125$ m) and along the measurement lines considering them as thin lines in y‐dimension (destination; $w_{d}=w_{y}-2b_{d}=0.875$ m, where $b_{d}=0.125$ m is the width of the border excluded from measurements at each end, along y‐dimension). Finally, dividing the total intensity by the length of the measurement line ($w_{d}$) gives the average intensity across the measurement line at a distance $x_{d}$ as

(7) $$I_{1}\left(x_{d}\right)=\frac{I_{0}\beta }{w_{d}2{\pi \alpha }^{2}}\int_{y_{d}=b_{d}}^{b_{d}+w_{d}}\int_{y_{s}=0}^{w_{y}}\int_{x_{s}=-w_{x}/2}^{w_{x}/2}e^{-\sqrt{{\left(x_{d}-x_{s}\right)}^{2}+{\left(y_{d}-y_{s}\right)}^{2}}/\alpha }{\mathrm{dx}}_{s}\;{\mathrm{dy}}_{s}\;{\mathrm{dy}}_{d},$$

The integrations above incorporate the contribution of each source point $\left(x_{s},y_{s}\right)$ to disease intensity at the destination point ($x_{d}$, $y_{d}$). In the integrals in Equation (7), we set $x_{s}=0$ at the center of the inoculation area and $y_{s}=0$, $y_{d}=0$ at the edge of the plot.

We fitted the one‐dimensional model Equation (6) and the two‐dimensional model Equation (7) to observed dispersal gradients to estimate the scale parameter α.

#### 2.2.2 Stripe rust

We analyzed a subset of data that corresponds to Hermiston 2002 and Madras 2002 trials (Cowger et al., 2005; Sackett & Mundt, 2005). Asexual spores of *P. striiformis* (urediniospores) were inoculated onto 1.53 m × 1.53 m squares within 6.1 m‐wide plots that were at least 100 m long in the downwind direction (Cowger et al., 2005). Disease severity in the measurement lines was measured visually as the percentage of leaf area covered by lesions (“disease severity” is a specific form of the more general term “disease intensity”, Madden et al., 2007).

We used the modified power‐law kernel

(8) $$\kappa_{p1}\left(r\right)=C_{k,p1}{\left(\lambda^{2}+r^{2}\right)}^{-\gamma /2}.$$

as defined by Equation (10) of Mikaberidze et al. (2016), because it describes disease gradients of stripe rust better than exponential or Gaussian kernels. Here, r is the distance between the source point and the destination point; $C_{k,p1}$ is the normalization factor, k=1,2 is the number of dimensions. At k=1, $C_{1,p1}=2\lambda^{\gamma -1}\Gamma \left(\gamma /2\right)/\left[\sqrt{\pi }\Gamma \left(\left(\gamma -1\right)/2\right)\right]$, where $\Gamma \left(\cdot \right)$ is the gamma‐function; and at k=2, $C_{2,p1}=\left(\gamma -2\right)/\left(2{\pi \lambda }^{2-\gamma }\right)$.

The kernel Equation (8) has the same basic properties as the modified power‐law kernel in Equation (5) (fat‐tailed, power‐law). This form was used by Mikaberidze et al. (2016) to analyze the same data and we decided to use the same form to enable an easier comparison. Similarly to Equation (5), γ is the shape parameter, λ is the scale parameter (set to λ = 0.762 m as in Mikaberidze et al., 2016).

Using Equation (8) and point source approximation (r=x, k=1), we computed the disease severity after dispersal at a distance x from the source

(9) $$I_{1}\left(x\right)=C_{1,p1}I_{0}\beta {\left(\lambda^{2}+x^{2}\right)}^{-\gamma /2}.$$

Then, we lifted the point source approximation and we computed the average disease severity in a measurement line at a distance $x_{d}$ from the middle of the source by substituting the kernel Equation (8) into Equation (1) and specifying the integrals according to the experimental design as

(10) $$I_{1}\left(x_{d}\right)=\frac{I_{0}\beta }{w_{d}}C_{2,p1}\int_{y_{d}=b_{d}}^{b_{d}+w_{d}}\int_{y_{s}=b_{s}}^{b_{s}+w_{x}}\int_{x_{s}=0}^{w_{x}}{\left(\lambda^{2}+{\left(x_{d}-x_{s}\right)}^{2}+{\left(y_{d}-y_{s}\right)}^{2}\right)}^{-\gamma /2}{\mathrm{dx}}_{s}\;{\mathrm{dy}}_{s}\;{\mathrm{dy}}_{d},$$

where $w_{x}=w_{y}=1.53$ m is the side length of the square source, $b_{s}=2.285$ m is the border area between the inoculation area and the edge of the plot, $w_{d}=3$ m is the length of each measurement line (y‐dimension), $b_{d}=1.525$ m is the width of the border excluded from measurements at each end (Figure 2b).

Following Sackett and Mundt (2005), and Mikaberidze et al. (2016), we performed a natural logarithmic transformation of observed disease gradients to avoid a disproportionate emphasis on the few large values at the beginning of the gradient, and excluded zeros from the log‐transformed data. Accordingly, we log‐transformed both the one‐dimensional model Equation (9) and the two‐dimensional model Equation (10). We then fitted both functions to log‐transformed disease gradients to estimate the shape parameter γ.

#### 2.2.3 Potato late blight

We analyzed a subset of data on dispersal of *P. infestans* (Gregory, 1968, Table III, unsprayed experiment). *P. infestans* zoospores were inoculated across a strip in the middle of each experimental plot (orange area in Figure 2c). Plots were 8.5 m wide and over 60 m long. Rows of potato plants were oriented along the plot length (along the x‐axis). Disease severity was measured as numbers of late blight lesions counted visually on all leaves belonging to two adjacent potato stems chosen in every row at several distances from the source: from 1 m up to 30 m.

When describing the experiment, Gregory (1968) did not report the widths of the inoculation areas and the measurement lines (along the x‐axis), but only stated that they spanned five plants and two plants along a row, respectively. The paper also did not specify the location of the origin from which the distances between the source and the measurement locations were determined. We inferred these details based on reasonable assumptions. First, we assumed the typical distance between plants along the row to be 0.38 m (15 inches). Based on that, we computed the width of the inoculation area as 1.9 m and the width of a measurement line as 0.76 m. Second, we assumed that the distances were measured from the edge of the inoculation area (Figure 2c), as otherwise the first measurement line would overlap with the inoculation area.

The power‐law function

(11) $$\kappa_{p2}\left(r\right)=r^{-\gamma }$$

used to describe disease gradients by Gregory (1968) is not a kernel. Nevertheless, we used it in the analysis as if it were a kernel so that the results are comparable with the estimates obtained by Gregory (1968).

Under point source approximation, we used Equation (11) to compute the disease severity after dispersal at a distance r=x from the edge of the source

(12) $$I_{1}\left(x\right)=I_{0}\beta x^{-\gamma }$$

Next, we used the spatially explicit approach and computed the disease severity (averaged over the length of a measurement line) at a distance $x_{d}$ from the source by substituting Equation (11) into Equation (1) and specifying the integrals according to the experimental design

(13) $$I_{1}\left(x_{d}\right)=\frac{I_{0}\beta }{w_{d}}\int_{y_{d}=0}^{w_{d}}\int_{y_{s}=0}^{w_{y}}\int_{x_{s}=-w_{x}}^{0}{\left(\sqrt{{\left(x_{d}-x_{s}\right)}^{2}+{\left(y_{d}-y_{s}\right)}^{2}}\right)}^{-\gamma }{\mathrm{dx}}_{s}\;{\mathrm{dy}}_{s}\;{\mathrm{dy}}_{d},$$

where $w_{x}=1.9$ m and $w_{y}=8.5$ m are the dimensions of the inoculation area. Both the inoculation area and the measurement lines spanned the entire width of the plot in y‐dimension ($w_{y}=w_{d}$). Since the function in Equation (11) cannot be normalized, there is no normalization factor in Equation (12) and Equation (13). For this reason, the prefactor $I_{0}\beta$ in Equation (12) and Equation (13) has no biological relevance.

We performed the natural logarithmic transformation of observed disease gradients and excluded zeros from transformed data. Accordingly, we log‐transformed both the one‐dimensional Equation (12) and two‐dimensional Equation (13). Then, we fitted the two functions to observed disease gradients to estimate the shape parameter γ.

#### 2.2.4 Data analysis

The fitting was implemented in Python 3.7 using packages numpy (v. 1.17.3, Harris et al., 2020), scipy (v. 1.3.1, Virtanen et al., 2020) and lmfit (v. 1.0.1, Newville et al., 2014). We used least squares optimization via the function ‘Model.fit’ of the package lmfit (Levenberg–Marquardt or Trust Region Reflective algorithm, function scipy.optimize.least_squares). The same method was used in all analyses of the experimental data and in numerical simulations. Brute force search was used with experimental data to achieve reasonable starting values for the least squares optimization.

We used the estimates of the kernel parameters for septoria tritici blotch and stripe rust to quantify the characteristic scales of dispersal by computing medians ($r_{50}$) and 90th percentiles ($r_{90}$) of dispersal distance kernels (Nathan et al., 2012). We computed the two percentiles numerically by solving the equation $2\pi \int_{0}^{r_{L}}r\kappa_{i}\left(r\right)\;\mathrm{dr}=0.01L$ with respect to $r_{L}$ at L=50,90. Here, $\kappa_{i}\left(r\right)$ is the dispersal kernel function, where i=e,p1; e stands for the exponential kernel (Equation (3) at k=2) and p1 stands for the modified power‐law kernel (Equation (8) at k=2).

## 3 RESULTS

### 3.1 Results of data analysis

In all three cases, the spatially explicit estimation (2D‐estimation) resulted in steeper dispersal kernels and shorter dispersal distances compared to the point source approximation (1D‐estimation), because the estimated kernel parameters differed between 2D‐ and 1D‐estimation (Table 2, Appendix S1: Figure S1.1). α‐estimate for septoria tritici blotch was lower by about 12% in 2D‐estimation compared to 1D‐estimation; γ‐estimate for stripe rust was higher by about 10%; γ‐estimate for potato late blight was higher by more than 30%.

**TABLE 2:** Comparison of kernel parameter estimates and associated percentiles of dispersal distance kernels between one‐ and two‐dimensional models (1D and 2D, respectively). The 1D‐estimates here correspond to the estimates presented in the earlier publications.

		Septoria tritici blotch	Stripe rust, Madras	Stripe rust, Hermiston	Late blight
1D	Parameter	α=0.151 m	γ=2.4	γ=2.5	γ=1.4
	$r_{50}$, m	0.25	4.2	3.0	–
	$r_{90}$, m	0.58	241	76	–
2D	Parameter	α=0.135 m	γ=2.6	γ=2.7	γ=1.9
	$r_{50}$, m	0.23	2.3	1.9	–
	$r_{90}$, m	0.52	35.3	20.4	–

*Note*: The parameter γ appears in the exponent of power‐law kernels, but the parameter α enters the denominator of the exponent in the exponential kernel. Hence, the parameter difference has the opposite effect on characteristic dispersal distances in septoria tritici blotch compared to the two other systems.

For septoria tritici blotch and stripe rust, we further investigated how the differences in kernel parameter estimates affect the characteristic spatial scales of dispersal, quantified by the 50th and 90th percentiles of dispersal distance kernels, $r_{50}$ and $r_{90}$. For septoria tritici blotch, a moderate reduction in the α‐estimate in 2D‐estimation compared to 1D‐estimation translated into a similarly moderate decrease in $r_{50}$ and $r_{90}$ (Table 2). In contrast, for stripe rust, a modest increase in the γ‐estimate in 2D‐ versus 1D‐estimation translated into a dramatic decrease in the spatial scales of dispersal (Table 2). In particular, for Madras dataset, 2D‐estimation resulted in a nearly two‐fold reduction of $r_{50}$ and a massive, almost seven‐fold reduction of $r_{90}$. Although we could not conduct this analysis with the power‐law function defined by Equation (11) since it cannot be normalized, based on the substantial difference in the γ‐estimates between 2D‐ and 1D‐estimation, we expect a comparably strong reduction in estimated characteristic spatial scales of dispersal for potato late blight too. Thus, the three plant pathogens disperse over substantially shorter distances according to more realistic 2D‐estimation compared to conventional 1D‐estimates.

### 3.2 Results of numerical simulations

Are the 2D‐estimates acquired above more accurate (i.e., closer to the true values) than 1D‐estimates? This is plausible, because the 2D‐estimation describes dispersal from spatially extended sources more realistically. However, we cannot answer this question definitively based on the analysis of experimental data alone, because we do not know the true values of dispersal kernel parameters. Here, we addressed this question via numerical simulations. We first simulated the dispersal process according to exponential, Gaussian and power‐law kernels with pre‐defined parameters. Then, we used both methods to estimate the kernel parameters and compared the two methods in terms of their estimation accuracy across a range of biologically plausible scenarios. Here, we summarize the key outcomes of these simulations, but describe them in more detail in Appendices A, B and C.

We started by conducting idealized simulations (Appendix S2): we assumed that sampling locations were points without spatial extent and that measured values accurately reflected true values. Here, the 2D‐estimation provided perfectly accurate estimates, while 1D‐estimation exhibited substantial errors. We analyzed how the errors in 1D‐estimates depend on the parameters of kernel functions and source sizes. We found that the errors become smaller for organisms with longer mean dispersal distances and when using smaller source sizes.

Next, we wanted to understand the origin of errors in 1D‐estimates. We considered different parts of the extended source area separately and analyzed how they ‘distort’ the parameter estimates when assuming a point source (Appendix S3). We found that different parts of the source generate different errors, depending on the kernel type and the location of the virtual point source (used in 1D‐estimate). For power‐law kernels, the errors in 1D‐estimation were particularly difficult to minimize even when we decreased the source size. None of the three ways we attempted to improve the accuracy of 1D‐estimation led to satisfactory outcomes for power‐law kernels (denser sampling, sampling further away from the source, or estimation of both λ‐ and γ‐parameters of the kernel). Hence, the 1D‐estimation has serious limitations for parameter estimation from extended sources while the 2D‐estimation is accurate.

Finally, we investigated both 1D‐ and 2D‐estimation in more realistic simulations where the design mimics more closely experimental design (Appendix S4). We found that sparser sampling and extension of measurement lines reduced the accuracy of both 1D‐ and 2D‐estimates. However, with sufficiently dense sampling the 2D‐estimates recovered their accuracy while the 1D‐estimates retained substantial errors in all cases.

Thus, we demonstrated that the effect of extended sources on the accuracy of the point source approximation is complex: the magnitude and even the direction of the error induced by the point source approximation depend in a non‐trivial manner on the type of the kernel, the scale of dispersal, the spatial configuration of the source and the properties of sampling. Thus, our outcomes discourage the use of the conventional point source approximation, as it provides accurate estimates only in exceptional cases that are hard to identify. Instead, our outcomes strongly support the use the spatially explicit method for estimating dispersal kernel parameters.

## 4 DISCUSSION

We devised a theoretical framework to estimate dispersal kernels from empirical dispersal gradients by incorporating the spatial extent of dispersal sources. We re‐analyzed existing dispersal gradients for three major plant pathogens and found that this spatially explicit approach provides considerably different estimates of dispersal kernels compared to the conventional point source approximation. Further, we demonstrated using numerical simulations that the spatially explicit approach yields more accurate estimates across a wide range of biologically plausible scenarios. Combining these two lines of evidence, we conclude that the three organisms disperse on average over substantially shorter distances compared to estimates from conventional modeling.

Similar spatially explicit approaches have been used in modeling studies to investigate dispersal in plants (Clark et al., 1999; Shaw et al., 2006) and plant pathogens (Rimbaud et al., 2018). However, such approaches are not adopted in the literature on empirical characterization of dispersal (e.g., not used in Werth et al., 2006; Skarpaas & Shea, 2007; Loebach & Anderson, 2018; Emsweller et al., 2018; Devaux et al., 2007). Also, Bullock et al. (2017) excluded dispersal gradients produced by line and area sources from their analysis, because these gradients could not be compared to dispersal gradients from point sources. However, dispersal kernels estimated using the spatially explicit approach presented here enable such comparisons, because the estimates are independent of specific experimental design. Hence, adoption of this methodology would provide a unifying framework to extract biological knowledge from experimental data on dispersal.

We demonstrated how to use this theory to extract more knowledge from existing dispersal datasets. The improved estimates can potentially enhance our understanding of ecological dynamics. For example, yellow rust pathogen *P. striiformis* has recently expanded its geographic range by adapting to higher temperatures (Milus et al., 2009). Here, we acquired more accurate estimates of *P. striiformis* dispersal kernels using the spatially explicit approach, whereby the characteristic dispersal distance $r_{90}$ is about seven times shorter compared to conventional estimates (Table 1). Thus, our results (combined with knowledge about other relevant biophysical processes) could enable a more accurate prediction of further range expansion of *P. striiformis* populations, which is likely to be slower than expected based on conventional estimates.

Similarly, using this method, a large proportion of other published dispersal gradients (e.g., Devaux et al., 2007; Emsweller et al., 2018; Loebach & Anderson, 2018; Skarpaas & Shea, 2007; Werth et al., 2006) can be re‐analyzed to improve our knowledge about spatial scales of dispersal. This could improve our capacity to predict shifts and expansions of species' geographic ranges, and sizes and compositions of plant communities. We demonstrated that depending on several factors (e.g., the functional form of the kernel and the spatial configuration of source/measurement locations), the improved estimates based on spatially explicit approach can result either in shorter or longer dispersal distances compared to conventional estimates. Accordingly, an improved prediction of rate of range expansions or shifts and sizes or compositions of ecological communities can go in either direction, which highlights the importance of acquiring more accurate estimates of dispersal.

We assumed isotropic dispersal in data analysis and simulations. However, anisotropic dispersal is common in nature (Soubeyrand et al., 2007) and the model can be extended to incorporate it. In this extended model, the probability of dispersal from a source point to a destination point will depend not only on the distance between the points, as in our case, but also on the direction from the source to the destination. Parameters of anisotropic dispersal kernels can then be estimated from measurements of dispersal gradients with the spatially explicit consideration of the source. Empirical data we analyzed characterized populations of passively dispersing plant pathogens. The methodology is applicable to plant and plant pathogen systems that have passive dispersal, but may not be applicable to characterize active dispersal, e.g., of vector‐borne plant viruses.

Based on the outcomes of our idealized simulations, it is tempting to propose simple rules of thumb about when the point source approximation provides reasonably accurate estimates of dispersal kernel parameters. This appears to be the case, for example, when the sources are sufficiently small and the spatial scale of dispersal is sufficiently large (Figure S2.2). However, in these idealized simulations, we neglected the spatial extent of measurement areas and limitations in the amount of sampling within these areas. When we considered these features in more realistic simulations, the outcomes revealed a non‐trivial influence of several factors (such as the functional form of the kernel, the spatial configuration of the source and the measurement locations as well as sample sizes) on the estimation accuracy. As a result, we are not able to provide simple rules of thumb regarding the validity of the point source approximation.

Instead, based on our results we suggest the following best practices to design future dispersal experiments. First, a proposed experiment should be simulated numerically over a range of plausible parameter values to decide whether the point source approximation is valid or the spatially explicit modeling should be used in the analysis. Second, aspects of experimental design can be optimized by doing further simulations in order to minimize costs while maximizing the estimation accuracy. These aspects include the size of the source, the spatial configuration of measurement areas (such as their sizes, shapes, and measurement distances), and sample sizes. In conclusion, we demonstrated how spatially explicit modeling can improve the analysis of existing dispersal data and optimize design of future dispersal experiments.

## CONFLICT OF INTEREST STATEMENT

The authors declare that they have no competing interests.

[Correction added on 21 April 2023, after first online publication: The data availability statement is updated in this version.].

## Supporting information


Appendix S1.

Data S1.

## Data Availability

Data and source code are provided in public repositories. Data: https://doi.org/10.5281/zenodo.7794187, source code: https://doi.org/10.5281/zenodo.6976556.
