# Supplementary material for: Spatially explicit ecological modeling improves empirical characterization of plant pathogen dispersal
Source: Plant Environ Interact. 2023 Apr 9;4(2):86–96. doi: 10.1002/pei3.10104 (PMC10243544; doi:10.1002/pei3.10104)
Supplement: Supplementary file 2 — Data S1. [file PEI3-4-86-s001.pdf]

Werth, S., Wagner, H.H., Gugerli, F., Holderegger, R., Csencsics, D., Kalwij, J.M., and Scheidegger, C., 2006. Quantifying dispersal and establishment limitation in a population of an epiphytic lichen. *Ecology*, 87(8), pp.2037–2046.

Zadoks, J.C. and Schein, R.D., 1979. *Epidemiology and plant disease management.* Oxford University Press Inc.

Plant-Environment Interactions - Supporting Information

Article title: Spatially explicit ecological modeling improves empirical characterization of plant pathogen dispersal

Authors: Petteri Karisto, Frédéric Suffert, and Alexey Mikaberidze

The following Supporting Information is available for this article:

- Appendix S1: Fitting empirical disease gradients
- Appendix S2: Idealized simulations
- Appendix S3: Contributions of individual points within the source
- Appendix S4: Simulations with a more realistic design

Appendix S1: Fitting empirical disease gradients

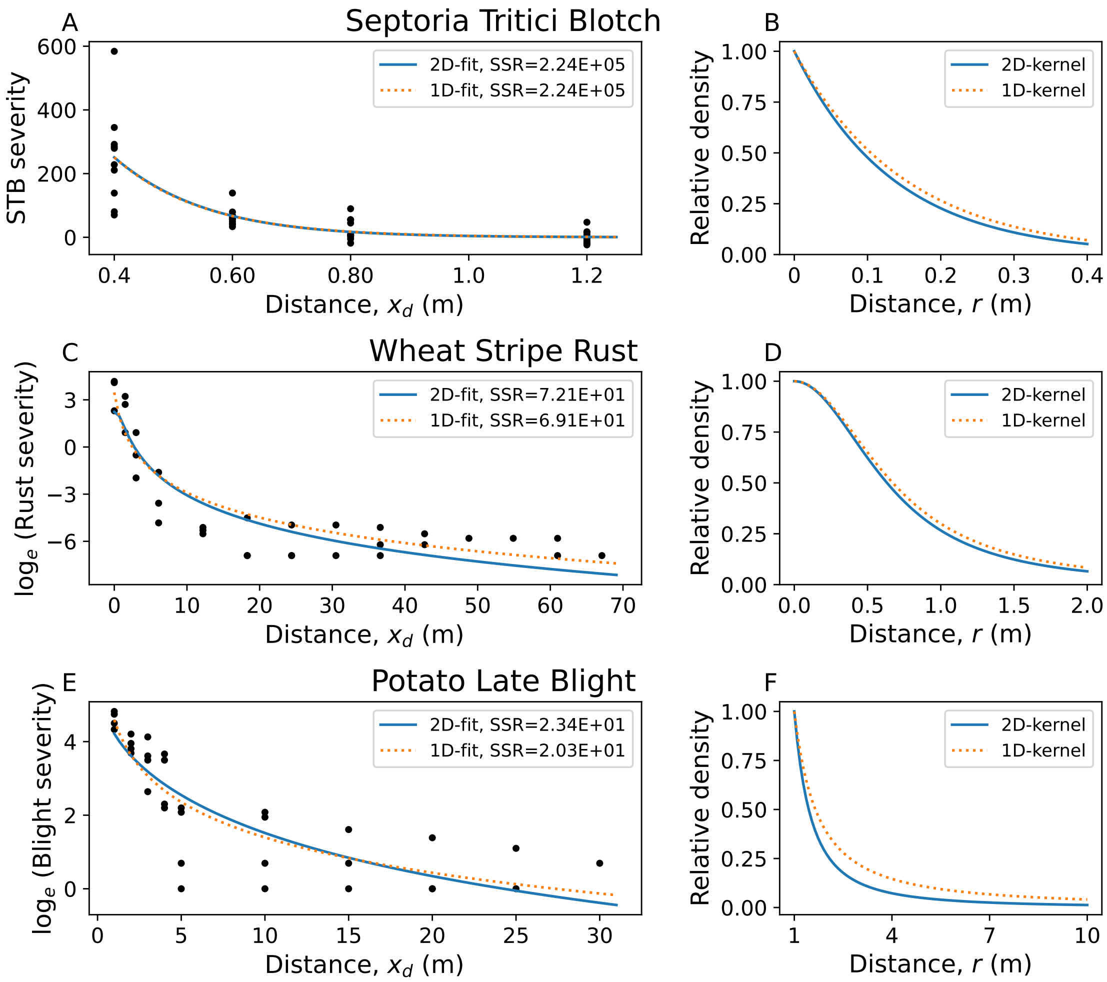

Figure S1.1: Estimation of dispersal kernels from empirical data. Left column: observed disease gradients (black circles) and the best-fitting models based on the spatially explicit approach ("2D-fit", solid blue curves) and the point source approximation ("1D-fit", dotted orange curves). The legends display the sums of squared residuals (SSR). Right column: the estimated dispersal kernels with best-fitting parameters according to the spatially explicit approach ("2D-kernel", solid blue curves) and the point source approximation ("1D-kernel", dotted orange curves). Kernels are normalized to start from one at $r = 0$ m for septoria tritici blotch and stripe rust but at $r = 1$ m for late blight, as the chosen power-law function diverges at zero.

Appendix S2: Idealized simulations

In the case studies in the main text, the spatially explicit approach (2D-estimation) resulted in substantially different estimates of dispersal kernels compared to the point source approximation (1D-estimation). Are these estimates more accurate, i.e., closer to the true values? This is plausible, because the 2D-estimation describes dispersal from spatially extended sources more realistically. However, we cannot answer this question definitively based on the analysis of experimental data alone, because we do not know the true values of dispersal kernel parameters. Here, we addressed this question via numerical simulations. We first simulated the dispersal process according to exponential, Gaussian and power-law kernels with pre-defined parameters. Then, we used both methods to estimate the kernel parameters and compared the two methods in terms of their estimation accuracy.

We started by conducting idealized simulations: the 2D-estimation provided perfectly accurate estimates, while 1D-estimation exhibited substantial errors. We analyzed how these errors depend on the parameters of kernel functions and source sizes. Further, in Appendix S3, we considered how different parts of the extended source area 'distort' the parameter estimates when assuming a point source. Finally, in Appendix S4, we examined a more realistic scenario that incorporated extended measurement areas (instead of measurement points) and a limited amount of sampling within the areas.

Methods

Which parameters did we estimate?

The exponential and Gaussian kernel functions have a single parameter: the scale parameter α that characterizes the spatial scale of dispersal (see Box 1) and this was the parameter we estimated. In contrast, the power-law kernel has two parameters: the scale parameter λ and the shape parameter γ both of which influence the spatial scale of dispersal. To make the estimation comparable between the three different kernels, we set λ to its true value and estimated only γ. Thus, we estimated a single parameter in each of the three dispersal kernel functions, which we call the "kernel parameter".

Design of the simulations

We simulated dispersal from a spatially extended source and sampled the resulting dispersal gradients in an idealized manner: we assumed that sampling locations were points without

any spatial extent and that the measured values accurately reflected the true values.

First, we fixed the source size (1 m × 1 m) and conducted simulations using different mean dispersal distances from 1 m to 50 m (in 1 m steps) to study the effect of the range of dispersal on the accuracy of the 1D-estimation. (Expressions for mean dispersal distances for each of the three kernels are given in Box 1 in the main text). Next, we simulated dispersal with a fixed mean dispersal distance (20 m), but varied the size of the square source from 1 m × 1 m to 30 m × 30 m (in 1 m steps) to study the effect of the source size.

We derived the number of dispersed individuals from Eq. (1) by considering the destination as a point and using a square source area:

$$N_1(x_d, y_d) = N_0 \int_{y_s=-w_y/2}^{w_y/2} \int_{x_s=-w_x/2}^{w_x/2} \kappa_i(r) dx_s\, dy_s, \tag{S2.1}$$

where the origin of the coordinates is set to the center of the square source, $r = \sqrt{(x_s - x_d)^2 + (y_s - y_d)^2}$ is the distance between the source point (x_s, y_s) and the destination point (x_d, y_d), $w_y = w_x$ is the side length of the square source, and $\kappa_i(r)$ is the dispersal kernel function, where $i = e, g, p$ stands for exponential, Gaussian and power-law kernels, respectively (we used Eqs. (3)-(5) in Box 1, setting $k = 2$, and setting $\lambda = 0.5$ m for the power-law kernel). We assumed in Eq. (S2.1) that the population of N_0 dispersing individuals was distributed uniformly across the spatial extent of the source.

We generated the dispersal gradients by evaluating the number of dispersed individuals $N_1(x_d, y_d)$ according to Eq. (S2.1) at $y_d = 0$ and a number of different values of x_d, starting from 10 cm away from the edge of the source in steps of 10 cm until reaching the point where the number of dispersed individuals was less than 1% of that of the first measurement point (dotted lines in Fig. S2.1A and S2.1B).

The kernel parameter was then estimated from simulated data (i) using a two-dimensional dispersal kernel and incorporating the spatial extent of the source according to Eq. (S2.1) with the kernel functions given by Eqs. (3)-(5), setting $k = 2$; and (ii) using the corresponding one-dimensional dispersal kernels (setting $k = 1$), where we set $r = x_d$ in the expressions for the kernel functions i.e., considering a virtual point source located at the center of the actual source area. In Appendix S3, we used a similar analysis to explore the case when the virtual point source is located at the edge of the source area, rather than its center, as that is sometimes assumed in dispersal experiments, for example, in the potato late blight case study above presented in the main text (Gregory, 1968).

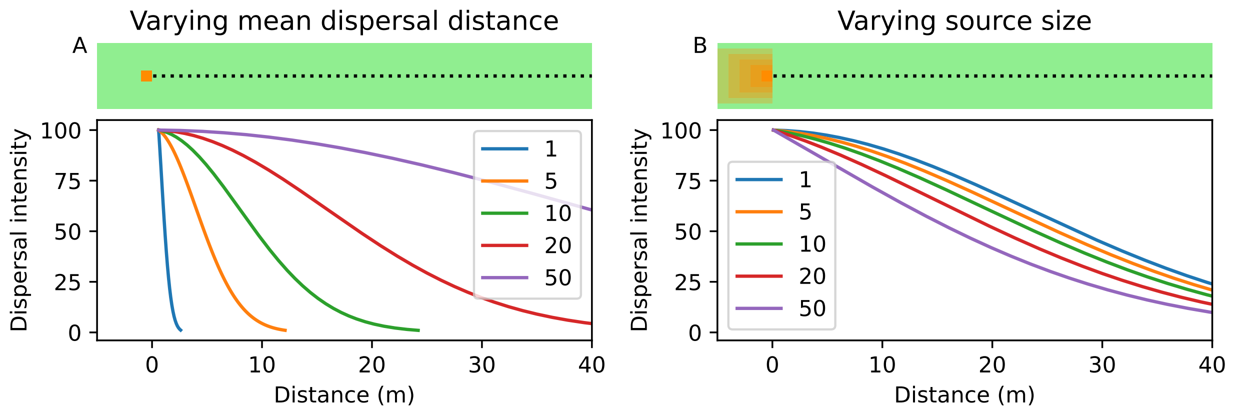

Figure S2.1: Plot designs and example gradients in the idealized simulations with the Gaussian kernel Eq. (4). Measurements were taken along the dotted line every 10 cm. A) Mean dispersal distances were varied from 1 m to 50 m, while the size of the square source was fixed to 1 m×1 m. B) Source side lengths were varied from 1 m to 50 m, while the mean dispersal distance was fixed to 20 m.

Results and discussion

How does the accuracy of the point-source approximation depend on the kernel parameter and the source size?

In the idealized simulations, when we incorporated the spatial extent of the source in the analysis (2D-estimation), the kernel parameter estimates were error-free in all cases (up to very small errors inherent in numerical computation). In contrast, the estimation under the point source approximation (1D-estimation) resulted in substantial errors. We investigated how these errors change when we vary the true values of mean dispersal distances (Fig. S2.2A) and when we vary the source size (Fig. S2.2B). The 1D-estimates of the kernel parameter become more accurate at longer mean dispersal distances for each of the three kernel functions (Fig. S2.2A). Similarly, the estimates become more accurate when using smaller sources (Fig. S2.2B).

1D-estimates of the power-law kernel parameter behave differently compared to the two other kernel functions. First, for both exponential and Gaussian kernels, the errors approach zero when the mean dispersal distance is increased, but for the power-law kernel, the errors appear to approach a substantially positive value (cf. green curve with blue and orange curves in Fig. S2.2A). Second, estimation errors increase with the size of the source much faster in the case of the power-law kernel compared to exponential or Gaussian kernels (cf. green curve with blue and orange curves in Fig. S2.2B).

In all three kernel functions, the point-source approximation leads to an overestimation of

their respective kernel parameters (all errors have a positive sign in Fig. S2.2A,B). However, the mean dispersal distances are overestimated for exponential and Gaussian kernels but underestimated for the power-law kernel under the point-source approximation (see below).

To conclude, when we estimate the kernel parameter under the point source approximation, the estimation accuracy increases for organisms with longer mean dispersal distances and when using smaller source sizes. Thus, in this idealized scenario, the estimation of kernel parameters under the point-source approximation could reach any desired level of accuracy by using a sufficiently small source.

Accuracy of estimation of mean dispersal distances

We also estimated the mean dispersal distances based on the estimates of kernel parameters and investigated how errors of these estimates depend on the true values of mean dispersal distance (Fig. S2.2C) and on the source size (Fig. S2.2D).

When dispersal is governed by exponential or Gaussian kernels, increasing the mean dispersal distance leads to a more accurate estimation of the kernel parameter. This translates into more accurate estimation of the mean dispersal distance (cf. blue curves and orange curves between Fig. S2.2A and S2.2C). In contrast, when dispersal is governed by the power-law kernel the increased accuracy of estimation of the kernel parameter does not translate into more accurate estimates of the mean dispersal distance. Instead, the error in estimates of the mean dispersal distance grows as a function of the true value of the mean dispersal distance (Fig. S2.2C) and as a function of the source side length (Fig. S2.2D). This seemingly counter-intuitive relationship follows from the expression of the mean dispersal distance for the power-law kernel (Box 1) $\bar{r}_p = 2\lambda/(\gamma - 3)$. To achieve longer mean dispersal distances in Fig. S2.2A,C, the true value of the kernel shape parameter γ needs to become closer to the critical value of three, where the mean dispersal distance experiences a singularity. For this reason, even decreased errors in the estimates of the kernel parameter γ translate into larger errors in the estimates of the mean dispersal distance $\bar{r}_p$.

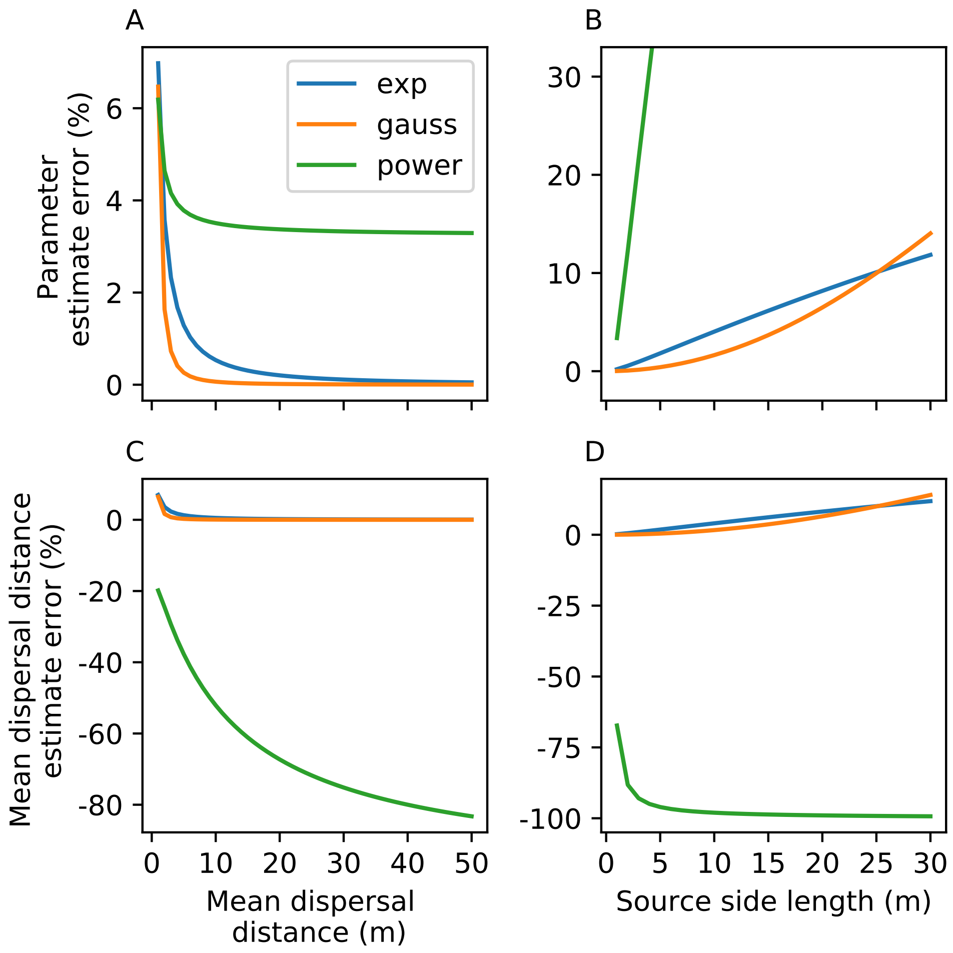

Figure S2.2: Effect of the mean dispersal distance ($\bar{r}$ varied, left column) and source size ($\bar{r} = 20\,\mathrm{m}$, right column) on the accuracy of the point source estimate (assuming a point source at the center of the actual square source). The error in the kernel parameter estimates (upper row) and in the estimated mean dispersal distance (lower row) is shown as a function of true mean dispersal distance and source size. Simulation design corresponds to Fig. S2.1A,B. Accuracy of estimation improves when considering organisms with longer mean dispersal distances and when using smaller sources. Parameters: $\beta I_0 = 10000$ (arbitrary), $\lambda = 0.5\,\mathrm{m}$, $\alpha_e = \bar{r}/2$, $\alpha_g = \bar{r}\sqrt{2/\pi}$, $\gamma = 3 + 2\lambda/\bar{r}$.

Appendix S3: Contributions of individual points within the source

To better understand the patterns we observed in simulations above, we studied in more detail how the errors in the estimation of kernel parameters (and estimated mean dispersal distance) arise under the point-source approximation. We decomposed the overall error associated with the point-source approximation into its more basic ingredients: we considered how each displacement of the point source from its location at the center of a square source to a different location within the source area contributes to the error in the estimated kernel parameter.

Methods

For this purpose, we considered a square $1\,\text{m} \times 1\,\text{m}$ source and a dispersing population with $2\,\text{m}$ mean dispersal distance. We simulated dispersal from each individual point in a $2\,\text{cm} \times 2\,\text{cm}$ square grid within the source area for each of the three kernel functions (setting $\lambda = 0.5\,\text{m}$ for the power-law kernel). In this way, we generated the dispersal gradients produced by individual points across the area of the source according to Eq. (S2.1). Then, we estimated the kernel parameters (as described above) and the associated mean dispersal distances from dispersal gradients originating from each individual point within the source but assuming that the source was a point located at the center of the source area. We then recorded whether the estimates of kernel parameters were higher or lower than their true values (Fig. S3.1).

We also investigated the effect of assuming a virtual point-source at the edge of the source area, rather than at the center. Also, we considered in more detail the case of power-law kernels, since it yielded qualitatively different results compared to exponential or Gaussian kernels.

Results and discussion

How is the accuracy of the point-source approximation influenced by the spatial extent of the source?

When dispersal is governed by an exponential kernel, we observe a simple pattern. Points within the source area along the continuation of the line on which measurements are conducted (the horizontal line drawn through the center of the source area in Fig. S3.1A) yield

accurate estimates, whereas any other points within the source area result in an overestimation of the kernel parameter. This follows from the memorylessness property of exponential kernels (Box 1): dispersal gradients produced by the points along this line all have the same shape and this shape corresponds exactly to the shape of the gradient produced by the point source at the center of the source. Any displacement of the source point from this line leads to a modification of the gradient shape (more specifically, to a flattening of the gradient) and hence an overestimation of the kernel parameter.

Gaussian kernel exhibits a similarly simple pattern that stems from its separability property (Box 1). In this case, the line drawn through the center of the source perpendicularly to the line of measurement locations has a special significance (Fig. S3.1B). Each point source on this line produces a gradient of the same shape as the point source at the center of the source area, leading to an accurate estimation of the kernel parameter. Any other points within the source area produce either steeper gradients (left half of the source area) or flatter gradients (right half of the source area). This leads to an underestimation (blue area in Fig. S3.1B) or an overestimation (red area in Fig. S3.1B) of the kernel parameter.

The power-law kernel is neither memoryless nor separable and hence we observe a more complex pattern: points within a circular area adjacent to the measurement locations lead to an overestimation of the kernel parameter, while all other points within the source area result in its overestimation (Fig. S3.1C).

Based on these patterns in Fig. S3.1, we can now understand why the point-source approximation leads to an overestimation of kernel parameters for all three kernel functions in Fig. S2.2. The overall gradient produced by the square source is a weighted sum over individual gradients produced by each of the point sources comprising the spatially-extended source (according to Eq. (S2.1)). Under the point-source approximation, we estimate the kernel parameter by fitting the one-dimensional kernel function to this overall gradient. Hence, the error of this estimation can be understood on the basis of the errors resulting from displacing the point source from the center to a different location within the source area.

For the exponential kernel, every such displacement produces a dispersal gradient, which when fitted by a one-dimensional kernel results in an overestimation of the kernel parameter: the whole area of the source is red in Fig. S3.1A, except for the black horizontal line. Therefore, fitting of the one-dimensional kernel function to the overall dispersal gradient will also lead to an overestimation of the kernel parameter.

In the case of the Gaussian kernel, one half of the displacements of the point source from the center lead to an overestimation, while the other half leads to an underestimation of the kernel parameter (Fig. S3.1B). However, when we compute the overall gradient as a weighted sum over individual gradients produced by each of the points sources, and estimate the kernel

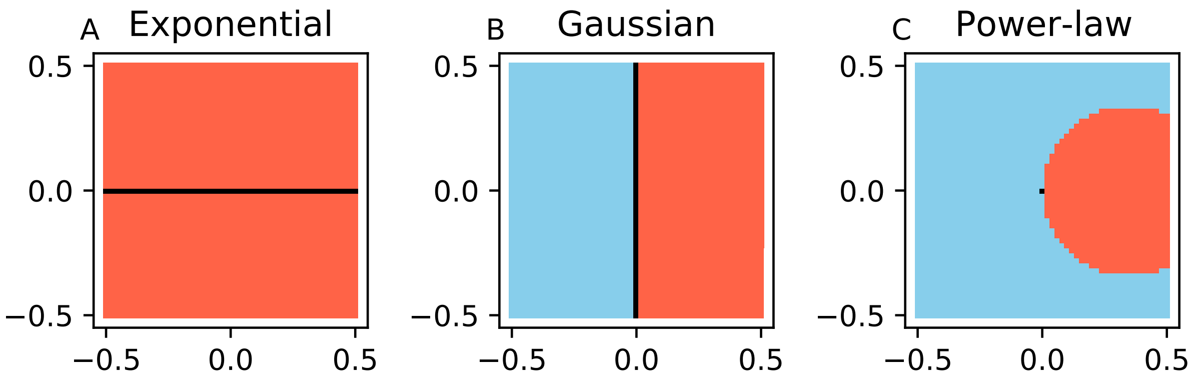

Figure S3.1: Contributions of individual points within the 1 m × 1 m source area to the errors in the kernel parameter estimates. Here, we considered a virtual point-source at the center of the actual square source area. For each point within the source area (across a 2 cm × 2 cm grid) the color shows, whether the point contributes to an underestimation (blue) or an overestimation (red) of the kernel parameter, or represents an accurate estimate (black). Measurements are conducted on the right side of the source area as in Fig. S2.1A. Parameters: $\beta I_0 = 1$ (arbitrary), $\bar{r} = 2\,\mathrm{m}$, $\lambda = 0.5\,\mathrm{m}$, $\alpha_e = \bar{r}/2$, $\alpha_g = \bar{r}\sqrt{2/\pi}$, $\gamma = 3 + 2\lambda/\bar{r}$.

parameter under the point-source approximation, the kernel parameter is overestimated. This is because the point sources that lead to an overestimation (the right half of Fig. S3.1B) contribute with higher weights, since they are closer to the measurement locations.

In the case of the power-law kernel, a larger part of the source area corresponds to displacements of the point source leading to an underestimation of the kernel parameter (the blue region outside of the red circular area in Fig. S3.1C). Yet again, the point sources that lead to an overestimation of the kernel parameter (those within the red circular area in Fig. S3.1C) make disproportionately large contributions since they are closer to the sampling locations. For this reason, the fitting of the one-dimensional kernel function to the overall gradient generated by the whole source area leads to an overestimation of the kernel parameter (Fig. S2.2).

Thus, by analyzing the effects individual displacements of the point source from the center of the source area, we gained a deeper understanding of how the point source approximation produces errors in estimates of dispersal kernel parameters.

Effect of individual points on the estimated mean dispersal distance

Figure S3.1 showed how displacements of the point source from its location at the center of a square source to a different location within the source area contribute to the error in the estimated kernel parameter. Here, we present an analogue of Fig. S3.1 that considers the

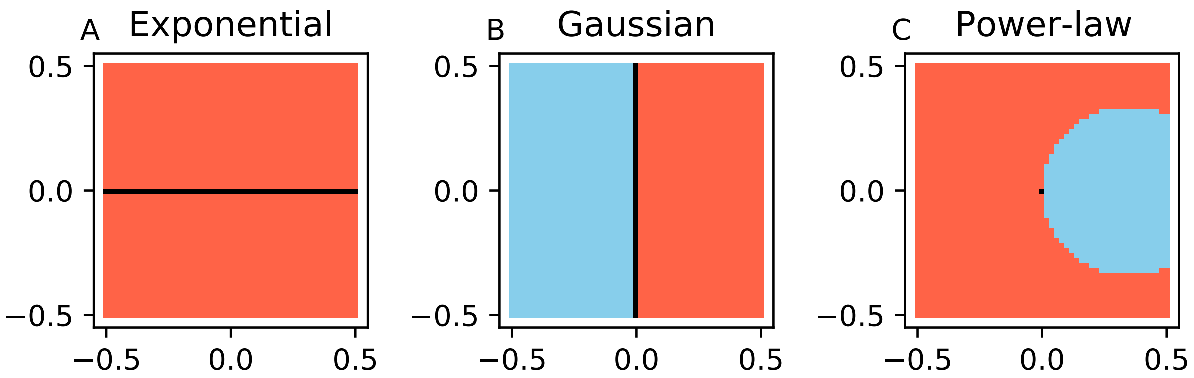

Figure S3.2: Contributions of individual points within the source area to the errors in the estimated mean dispersal distances $\bar{r}$. Here, we considered a virtual point source located at the center of the actual square source area. For each point within the source area (across a 2 cm × 2 cm grid) the color shows whether the point contributes to an underestimation (blue) or an overestimation (red) of the mean dispersal distance, or represents an accurate estimate (black). Measurements are conducted on the right side of the source area. Parameters: $\beta I_0 = 1$ (arbitrary), $\bar{r} = 2\,\mathrm{m}$, $\lambda = 0.5\,\mathrm{m}$, $\alpha_e = \bar{r}/2$, $\alpha_g = \bar{r}\sqrt{2/\pi}$, $\gamma = 3 + 2\lambda/\bar{r}$.

estimation of the mean dispersal distances in Fig. S3.2. Since the mean dispersal distance is proportional to the kernel parameter α for exponential and Gaussian kernels (Box 1), we observe the same patterns in the sign of the error when estimating the kernel parameter and the mean dispersal distance (cf. Fig. S3.1A,B with Fig. S3.2A,B). In contrast, in the case of the power-law kernel, the mean dispersal distance, $\bar{r}_p$, has an inverse relationship with the kernel parameter γ (Box 1). For this reason, the sign of the error in the dispersal distance estimates reverses compared to the sign of the error in the kernel parameter estimates (cf. Fig. S3.1C with Fig. S3.2C).

Virtual point source at the edge of the source area

We observed above that different parts of the area source can contribute differently to the distortion of the estimated kernel. Hence, it is plausible that moving the virtual point source (used in 1D-estimation) to different parts of the actual source area can affect the estimates.

In Appendix S2, we investigated estimation errors associated with the point source approximation considering a virtual point source located at the center of the square source area. Here, we conducted a similar analysis but considered a virtual point-source located at the edge of the source area that is closer to measurement locations. This change did not affect the overall pattern in the estimation accuracy of the kernel parameter. The estimates of the kernel parameter become more accurate when considering populations with longer mean

dispersal distances and when using smaller sources (Fig S3.3A, S3.3B). However, whether the mean dispersal distances are underestimated or overestimated, depends on the kernel function: they are underestimated for Gaussian kernels, but overestimated for exponential and power-law kernels (Fig S3.3C, S3.3D). Hence, for Gaussian and power-law kernels, the error changes its sign when we move the virtual point source from the center to the edge of the source area.

We can understand this change by comparing Fig. S3.2 and Fig. S3.4. In Fig. S3.2 the points to the right of the middle are closer to the measured gradient and hence have a more prominent effect on the shape of the overall gradient. Therefore, the right-hand side (red) area in Fig. S3.2B and the blue circular area in Fig. S3.2C contribute to the overall gradient with larger weights and result in the over estimation of the mean dispersal distance for the Gaussian kernel but underestimation for the power-law kernel (as seen in Fig. S2.2C, D). However, when we move the virtual point source to the edge of the source area, the measurements of the gradient start closer to the virtual point source point and those above mentioned areas of the source no longer contribute to the gradient. Hence, the sources in Fig. S3.4 match with the left halves of the sources in Fig. S3.2 and the dispersal distance is underestimated for the Gaussian kernel but overestimated for the power-law kernel.

For the power-law kernel, the kernel parameter is substantially underestimated across the whole range of parameters: green curves in Fig. S3.3A, S3.3B correspond to negative errors. For this reason, the kernel parameter estimates are in the range $\gamma < 3$ (while the true values are in the range $\gamma > 3$), leading to an infinite mean dispersal distance, $\bar{r}_p$ (this is a mathematical property of power-law kernels; see Box 1).

We conclude that it is difficult to guess *a priori* how the accuracy of estimation of the kernel parameters and mean dispersal distances would be affected by specific extensions of the source area or positioning of the virtual point source within the source area. Therefore, we are not able to provide a simple rule of thumb regarding the validity of the point-source approximation. Thus, using the spatially explicit approach is recommended to achieve more accurate estimation of dispersal kernel parameters.

Estimation of power-law kernel parameters is problematic under the point-source approximation

In Figure S2.2A, the error of the kernel parameter estimate approached zero quite fast for exponential and Gaussian kernels as we increased the mean dispersal distance. In contrast, for the power-law kernel, after an initial decline the error approached a constant value of around 3% at long mean dispersal distances. We wanted to better understand the reasons behind this qualitative difference and to investigate whether we could further reduce this remaining

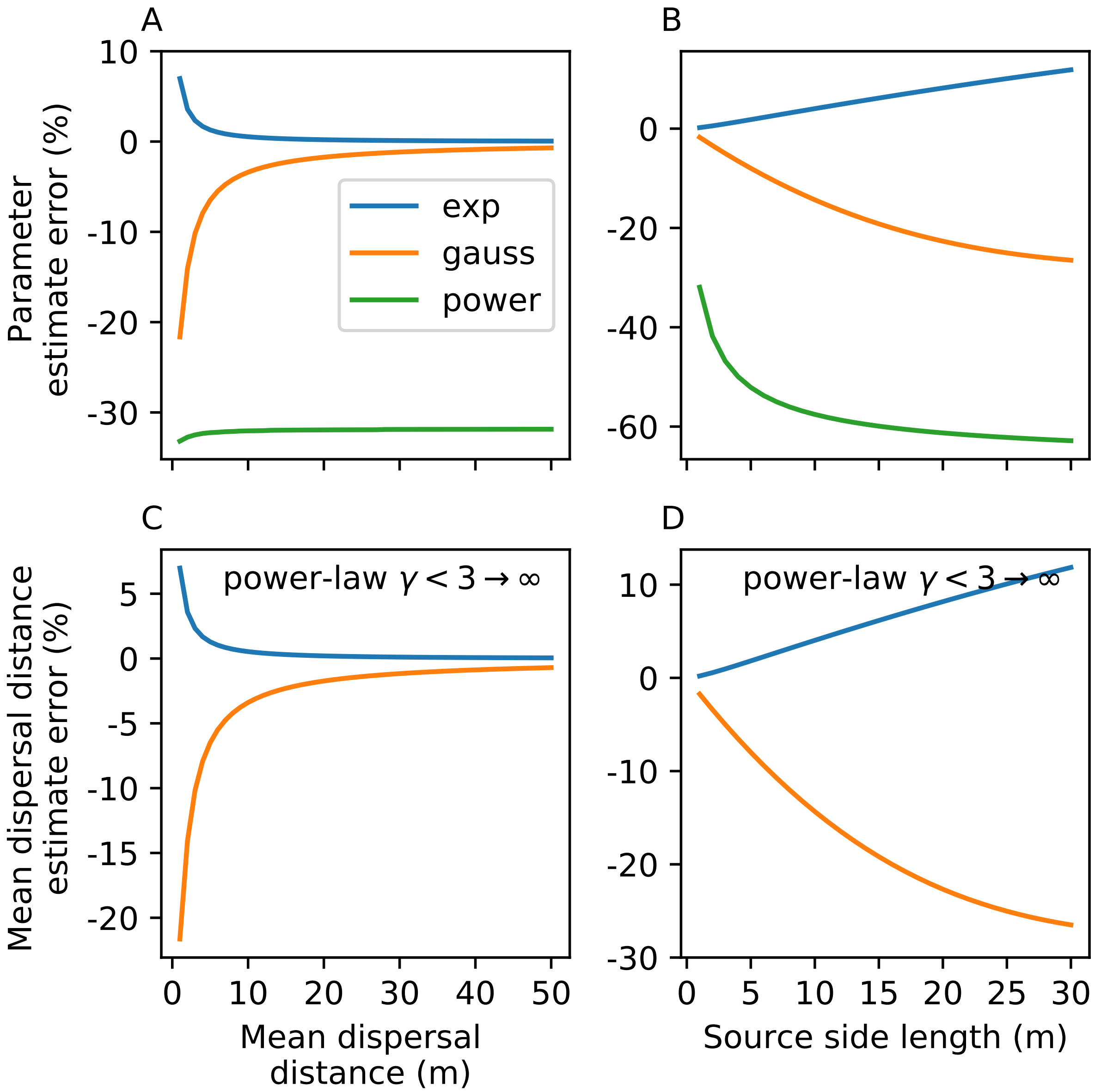

Figure S3.3: The accuracy of the point-source approximation depends on the true values of mean dispersal distances (left column) and on the source size (right column). Simulation design is the same as in Fig. S2.2, the only difference is that here a virtual point source is at the edge of the square source area, not at the center. Parameter estimates become more accurate when considering organisms with longer mean dispersal distances and when using smaller sources. Parameters: see Fig. S2.2.

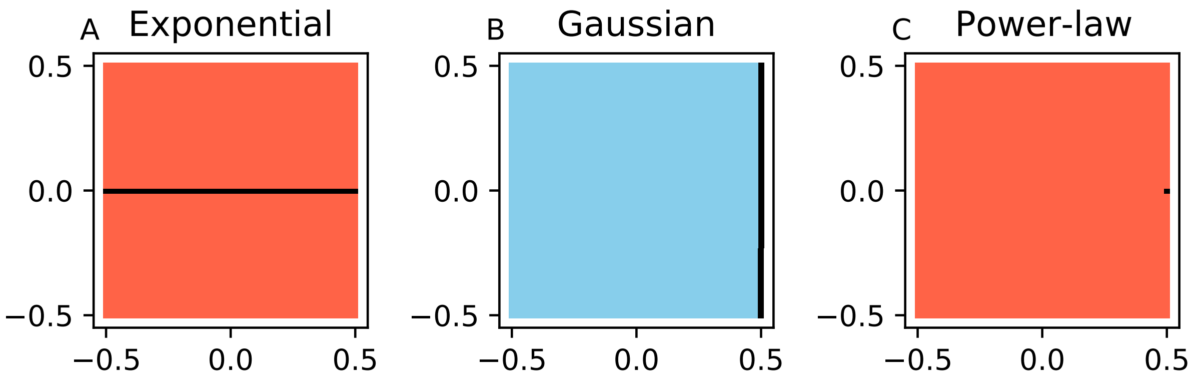

Figure S3.4: Contributions of individual points within the source area to the errors in the estimates of mean dispersal distances, $\bar{r}$. Here, we considered a virtual point source located at the edge of the actual square source area. For each point within the source area (across a 2 cm × 2 cm grid) the color shows whether the point contributes to an underestimation (blue), or an overestimation (red) of the mean dispersal distance, or represents an accurate estimate (black). Measurements are conducted on the right side of the source area. Parameters: see Fig. S3.2.

error. For this purpose, we formulated three hypotheses and tested them by modifying the simulation and fitting procedures. Each hypothesis assumes a specific reason behind the impaired accuracy of estimation of the power-law kernel parameter: (i) insufficiently dense sampling of the dispersal gradient (i.e., insufficient number of measurement lines within the fixed length of the measured gradient); (ii) insufficient capturing of the tail of the dispersal gradient; and (iii) fitting only the shape parameter γ of the power-law kernel, while setting the scale parameter λ to a constant value.

To test the first hypothesis, we increased the density of measurement lines. To test the second hypothesis, we introduced additional measurements further away from the source. To test the third hypothesis, we set the scale parameter of the power-law kernel (λ) free to be estimated alongside with the shape parameter γ. In these simulations, we used a 1 m-by-1 m source and set the mean dispersal distance to 20 m as in Fig. S2.2.

Before introducing these modifications, we re-state the "baseline" results. As explained in Appendix S2, we used the 10 cm distance between adjacent measurement lines and continued sampling until we reached 1% of the intensity in the first measurement line. In this case, the kernel parameter estimate was $\gamma_{\text{est}} = 3.153$. This estimate exhibits a 3.37% overestimation compared to the true value $\gamma_{\text{true}} = 3.05$. The associated mean dispersal distance was estimated to be 6.54 m, a severe underestimation of the true value of 20 m. (Note how a small error in the parameter estimate translates into a substantial error in the estimate of the mean dispersal distance when γ is close to its critical value of 3.)

(i) Sample more densely. We reduced the distance between adjacent measurement lines from 10 cm to 1 cm, thereby introducing a ten-fold increase in the density of measurements. We also started measurements from 1 cm away from the edge of the source, instead of 10 cm originally. As a result, the error in estimation decreased slightly from 3.37% to 2.25% overestimation ($\gamma_{\text{est}} = 3.119$) and the estimated mean dispersal distance was now 8.4 m, also slightly closer to the true value of 20 m.

(ii) Sample further away from the source. We decreased the threshold to stop the sampling: now we included all points that had at least 0.1% of the intensity of the first sampling line (the original threshold was 1%). This led to the estimate $\gamma_{est} = 3.152$ (an overestimation of 3.36%) corresponding to 6.56 m estimated mean dispersal distance. This represented only a negligible improvement in the estimation accuracy.

(iii) Estimate λ-parameter together with γ-parameter. We fitted the parameter λ together with the parameter γ. This improved quality of the 1D fit, leading to the chi-squared value of 0.699 compared to 1.278 in the original fit. However, the error in the shape parameter estimate increased ($\gamma_{est} = 3.337$ (error: 9.33%)) and so did the error in the estimate of the mean dispersal distance (3.50 m; $\lambda_{est} = 0.585$).

Conclusion. We introduced modifications to the sampling and fitting procedures to test the three hypotheses above. In each of the three cases, we achieved relatively minor improvements either in the accuracy of parameter estimates or in the quality of fits. The largest improvement in the estimation accuracy was gained by the ten-fold increase in sampling density: this reduced the error from 3.37% to 2.25%. However, this improvement does not appear satisfactory considering the increased cost of denser sampling. When sampling further away from the source (thereby capturing regions further into the tail of the dispersal gradient), the improvement in the estimation accuracy was negligible, while the practical challenges in detecting dispersing populations at 0.1% of their highest density would likely be considerable. Finally, fitting both parameters simultaneously improved the quality of the fit, but impaired the accuracy of parameter estimation. Thus, the one-dimensional fitting under the point-source approximation has serious limitations for parameter estimation of power-law dispersal kernels. These limitations can be overcome by using the spatially explicit method: it provided more accurate estimates in every case of our simulated dispersal experiments.

Appendix S4: Simulations with a more realistic design

We considered a scenario that incorporated key features of real dispersal experiments: not only a spatially-extended source, but also a spatially-extended destination and a limited sampling density.

Methods

As we did earlier in Appendix S2, we simulated dispersal governed by the three different kernel functions. The kernels and their parameters are given in Box 1 and Fig. 1: exponential, Gaussian and power-law kernels with mean dispersal distance $\bar{r} = 20\,\mathrm{m}$ ($\lambda = 20\,\mathrm{m}$ for power-law kernel). In addition to the square source area ($10\,\mathrm{m} \times 10\,\mathrm{m}$), here we also considered a circular source area (20 m diameter). Dispersal was simulated in a deterministic manner similarly to Appendix S2: we evaluated the number of dispersed individuals at different values of x_d, corresponding to distances of 10 m, 20 m, 30 m, 50 m, 70 m, 100 m and 150 m from the edge of the source that is adjacent to measurement lines (Fig. S4.1). In contrast to idealized simulations in Appendix S2, we also incorporated the spatial extent of destination areas: we considered the destinations as thin lines along the y-axis ("measurement lines", brown vertical lines in Fig. S4.1). To achieve this, we evaluated $N_1(x_d, y_d)$ according to Eq. (S2.1) for each value of x_d (i.e., for each measurement line) at five different values of y_d evenly spaced across the length $w_d = 40\,\mathrm{m}$ of the measurement line (filled black circles in Fig. S4.1). We also investigated the role of increasing the number of sampling points from five to 10, 40, and 160, and the role of shortening the measurement lines from 40 m to 20 m, 5 m, and 1 m presented in the later sections of the appendix.

In a typical dispersal experiment (such as the ones considered in "Analysis of empirical data" section of the main text), a number of measurements are conducted across measurement lines and their outcomes are recorded together with the value of x_d, but it is not practically feasible to also record the values of y_d. For this reason, an average is taken over all measurements conducted within each measurement line and these average values of N_1 versus x_d represent the measured dispersal gradients. We mimicked this measurement process here by computing an average over all N_1-values within each of the measurement lines.

We fitted the resulting dispersal gradients to models to estimate kernel parameters in the following way. First, the spatially explicit fitting was performed, whereby the function

$$N_1(x_d) = N_0 \int_{y_d=-w_d/2}^{w_d/2} \int_{y_s=-w_y/2}^{w_y/2} \int_{x_s=-w_x}^{0} \kappa_i(r) dx_s\, dy_s\, dy_d \tag{S4.1}$$

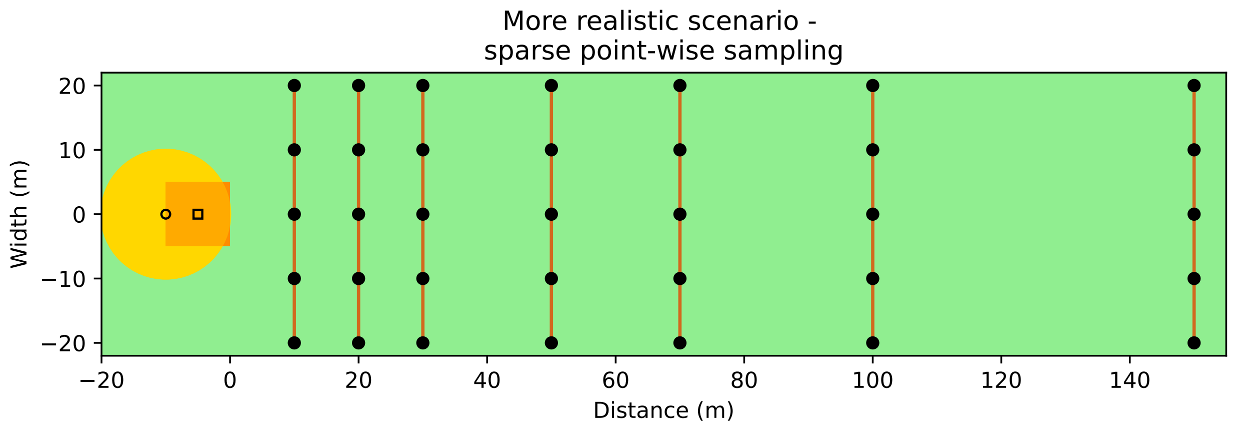

Figure S4.1: Design of simulations in a more realistic scenario, where we considered two source shapes (circle and square, orange) and sparse, point-wise sampling, where black circles show measurement locations (see text). Black open circle and square represent virtual point sources at the centers of the actual source areas.

that describes the number of dispersed individuals $N_1(x_d)$ versus x_d was fitted to simulated dispersal gradients. The meaning of variables and parameters in Eq. (S4.1) is the same as in Eq. (S2.1) in Appendix S2. Here, we also conducted integration over the length of the measurement line w_d (the outer integral in Eq. S4.1). In doing this, we assumed that the number of measurements taken within each measurement line was sufficiently high.

Second, the fitting under the point-source approximation was performed using one-dimensional kernels (Eqs. (3)-(5) in Box 1, setting $k = 1$), where we set $r = x_d + 5\,\mathrm{m}$ for the square source and $r = x_d + 10\,\mathrm{m}$ for the circular source in the expressions for the kernel functions, thereby considering a virtual point source located at the center of the actual source area.

Results and discussion

Realistic features of experimental design influence the estimation accuracy of the power-law kernel parameter under the point-source approximation

In these more realistic simulations, the kernel parameter estimates were not entirely accurate even when using the spatially explicit approach. Moreover, even in the idealized simulations, the error of the parameter estimate did not approach zero with increasing mean dispersal distances for the power-law kernel (green curve in Fig. S2.2A). We wanted to understand the reasons behind these remaining errors. For this purpose, we present here the outcomes of more detailed, additional simulations conducted using the power-law kernel. (Results of similar simulations for exponential and Gaussian kernels are available in the data repository, TBA).

Table S4.1: Estimation of kernel parameters from simulated dispersal data. The spatially explicit approach ("2D") provides more accurate estimates.

Source	Kernel	Parameter	1D	2D	True
Square	Exponential	α	11.34	10.26	10.0
	Gaussian	α	16.25	16.00	16.0
	Power-law	γ	4.27	4.83	5.0
Circle	Exponential	α	11.11	10.21	10.0
	Gaussian	α	16.77	16.00	16.0
	Power-law	γ	4.54	4.85	5.0

Note: "2D" and "1D" stand for two- and one-dimensional models, respectively; α is measured in m, γ is unitless. The scale parameter of the power-law kernel is set to $\lambda = 20\,\text{m}$.

Effect of sampling density on the estimation accuracy of the power-law kernel parameter

The inputs to the fitting procedure were means over all measurements within each measurement line, and the fitting assumed dense, uniform sampling (simulation design in Fig. S4.1). Hence, we hypothesized that higher sampling density within measurement lines would improve the accuracy of estimation. We were also interested in the sampling density required to achieve a desired level of accuracy.

We found that more dense sampling (achieved by increasing the number of sampling points while maintaining the size of the measurement lines) indeed led to more accurate estimates in both the spatially explicit approach (2D-estimation) and under the point-source approximation (1D-estimation). Importantly, at an increased number of sampling points, the 2D-estimates did approach their true value, while the 1D-estimates retained substantial errors (Tables S4.2 and S4.3).

Effect of the spatial extension of destinations on the estimation accuracy of the power-law kernel parameter

We hypothesized that the length of measurement lines (brown vertical lines in Fig. S4.1) introduced error in the estimation, as the number of dispersed individuals measured at the ends of the measurement lines (orange lines in Fig. S4.1) would be different compared to the one measured in the middle of the lines. We conducted simulations with shorter measurement lines to test this hypothesis and to determine whether shorter measurement lines would result in more accurate estimates.

We found that the estimates with shorter measurement lines were generally more accurate (Tables S4.2 and S4.3), thereby confirming the hypothesis. As we shortened the measurement

Table S4.2: Estimation of the power-law kernel parameter after simulating dispersal from a square source of 10 m × 10 m, with variable sampling density.

Measurement lines (m)	# of sampling points	1D	2D	True
1	5	5.06	5.00	5.0
1	10	5.06	5.00	5.0
1	40	5.06	5.00	5.0
1	160	5.06	5.00	5.0
5	5	5.03	4.99	5.0
5	10	5.04	5.00	5.0
5	40	5.04	5.00	5.0
5	160	5.04	5.00	5.0
20	5	4.72	4.90	5.0
20	10	4.78	4.96	5.0
20	40	4.81	4.99	5.0
20	160	4.82	5.00	5.0
40	5	4.27	4.83	5.0
40	10	4.35	4.92	5.0
40	40	4.41	4.98	5.0
40	160	4.42	5.00	5.0

Note: “2D” and “1D” stand for two- and one-dimensional models, respectively. The scale parameter of the power-law kernel is set to $\lambda = 20\,\text{m}$.

lines, 2D-estimates approached their true value, while 1D-estimates retained substantial errors.

Furthermore, we noticed that errors in the estimates of kernel parameters caused by the point source approximation in the more realistic scenario (Table S4.1, column “1D”) were consistent with the errors caused by the point-source approximation in the idealized simulations (where the destinations were considered as points) shown in Fig. S2.2 in the case of exponential and Gaussian kernels, but not in the case of the power-law kernel. For exponential and Gaussian kernels, the kernel parameters were overestimated both in the idealized simulations (Fig. S2.2) and in more realistic simulations Table S4.1 (column “1D”; more realistic simulations). In contrast, for the power-law kernel, the kernel parameter was overestimated in the idealized simulations, but underestimated in more realistic simulations. We wanted to find out why.

For this purpose, we focused on the subset of simulations with square source and five sampling points in each measurement line (these are extracted from Table S4.2 into Table S4.4). The kernel parameter was underestimated with 1 m and 5 m measurement lines (in agreement with idealized simulations) but overestimated with longer lines (column “1D” in Table S4.4). Hence, the extension of the destinations under the point-source approximation affected not

Table S4.3: Estimation of the power-law kernel parameter after simulating dispersal from a circle source of 20 m diameter, with variable sampling density.

Measurement lines (m)	# of sampling points	1D	2D	True
1	5	5.20	5.00	5.0
1	10	5.20	5.00	5.0
1	40	5.20	5.00	5.0
1	160	5.20	5.00	5.0
5	5	5.19	4.99	5.0
5	10	5.19	5.00	5.0
5	40	5.19	5.00	5.0
5	160	5.19	5.00	5.0
20	5	4.95	4.93	5.0
20	10	4.99	4.97	5.0
20	40	5.02	4.99	5.0
20	160	5.02	5.00	5.0
40	5	4.58	4.85	5.0
40	10	4.62	4.93	5.0
40	40	4.67	4.98	5.0
40	160	4.68	5.00	5.0

Note: “2D” and “1D” stand for two- and one-dimensional models, respectively. The scale parameter of the power-law kernel is set to $\lambda = 20$ m.

only the magnitude of the estimate error but also its sign. This explains why the sign of errors differed between the idealized and the more realistic simulations.

Table S4.4: Estimation of the power-law kernel parameter after simulating dispersal from a square source of 10 m × 10 m, varying the length of measurement lines (with five sampling points).

Measurement lines (m)	1D	2D	True
1	5.06	5.00	5.0
5	5.03	4.99	5.0
20	4.72	4.90	5.0
40	4.27	4.83	5.0

Note: “2D” and “1D” stand for two- and one-dimensional models, respectively. The scale parameter of the power-law kernel is set to $\lambda = 20$ m.

A similar change in the sign of the estimation error is also observed with circular source areas. Interestingly, in certain cases with circular source, elongated measurement lines and sparse sampling, the 1D-estimation resulted in more accurate estimates than the 2D-estimation. Note that the 1D-estimate happens to be more accurate than the 2D-estimate with 20 m measurement lines and 5 or 10 sampling points per line (column “1D” in Table

S4.3). In these cases, the estimation errors due to the extension of the source opposed the effect of the extension of measurement lines, which “accidentally” led to more accurate estimates since the two errors compensated for each other.

Summary

In Appendix S2, we conducted idealized simulations and found that errors in estimation of kernel parameters stem entirely from the point-source approximation. Here, we investigate a more realistic scenario that accounts for the fact that sampling locations are distributed over a finite area and sampling is conducted a limited number of times.

The spatially explicit estimation resulted in more accurate estimates of the kernel parameters in all cases (two different sources and three different kernels), but not entirely accurate (Table S4.1). We found that the reason for this remaining error was the finite number of sampling points within the extended destinations (i.e., along the measurement lines). When we increased the number of sampling points, the spatially explicit estimates approached their true value, while the estimates acquired under the point-source approximation retained a substantial error (see below, Tables S4.2, S4.3).

Interestingly, for exponential or Gaussian kernels, the point source approximation leads to overestimation of kernel parameters both here (Table S4.1, column “1D”) and in the idealized simulations in Fig. S2.2. In contrast, for the power-law kernel, the kernel parameter is underestimated here (Table S4.1, column “1D”), but overestimated in Fig. S2.2. Why?

To find out, we conducted additional simulations, where we varied the length of measurement lines (brown vertical lines in Fig. S4.1). We found that the error in the estimate of the power-law kernel parameter changes its sign when the measurement lines become sufficiently long (Table S4.4).

To conclude, the spatially explicit estimation remains substantially more accurate than the point source approximation in more realistic simulations. For the power-law kernel, the estimation error caused by the point source approximation depends in a non-trivial manner not only on the spatial scale of dispersal and the source size (Fig. S2.2), but also on the extent of measurement lines (Table S4.4). This makes it difficult to devise experimental designs that lead to a sufficiently accurate estimation when assuming a point source. Thus, we presented compelling evidence in favor of adopting the spatially explicit approach to estimation of dispersal kernel parameters.

References

Gregory, P., 1968. Interpreting plant disease dispersal gradients. *Annual review of phytopathology*, 6, p.189.
